# Permanent hypoparathyroidism following SARS-CoV-2 infection: a case report with two-year follow-up and literature review

**DOI:** 10.3389/fendo.2026.1712510

**Published:** 2026-05-25

**Authors:** Yizhi Zu, Hang Wang, Jinlin Wu, Cai Tang, Rui Han, Fang Fang

**Affiliations:** Department of Endocrinology and Metabolism, Chongqing Hospital of Traditional Chinese Medicine, Chongqing, China

**Keywords:** autoimmune, Hashimoto’s thyroiditis, hypocalcemia, hypoparathyroidism, SARS-CoV-2

## Abstract

Beyond its predominant respiratory involvement, Severe Acute Respiratory Syndrome Coronavirus 2 (SARS-CoV-2) infection has also been implicated in endocrine dysfunction. Hypocalcemia is common in Coronavirus Disease 2019 (COVID-19), yet the insufficient compensatory rise in parathyroid hormone (PTH) and its long-term outcomes remain poorly characterized. We describe a young adult Chinese woman with no history of neck surgery who developed severe symptomatic hypocalcemia shortly after acute SARS-CoV-2 infection. Laboratory evaluation revealed inappropriately low PTH levels with concomitant hyperphosphatemia, consistent with hypoparathyroidism (HypoPT). Although baseline calcium/PTH data prior to infection were unavailable, the regular calcium and vitamin D supplementation, along with serial monitoring over more than two years, demonstrated persistently suppressed PTH secretion, supporting a diagnosis of permanent HypoPT. Thyroid imaging and antibody testing confirmed coexistent Hashimoto’s thyroiditis, raising the possibility of an autoimmune contribution. To contextualize this case, we reviewed 14 previously published reports of COVID-19-associated HypoPT, among which no cases originating from China were identified. Based on available data, it cannot be ruled out that SARS-CoV-2 infection may contribute to the development of new-onset HypoPT in addition to worsening pre-existing conditions. Young-onset patients without a history of surgery—particularly those whose hypocalcemia severity does not clearly parallel the severity of infection—appear to have a higher likelihood of subsequent persistent dysfunction, though this observation remains preliminary. This case, together with the literature, underscores the need for extended follow-up in patients presenting with hypocalcemia and blunted PTH responses after COVID-19. Comprehensive autoimmune evaluation and, where appropriate, genetic testing should be considered to clarify etiology and guide long-term management.

## Introduction

The Coronavirus Disease 2019 (COVID-19) pandemic caused by Severe Acute Respiratory Syndrome Coronavirus 2 (SARS-CoV-2) has created profound and multifaceted health challenges worldwide. While the virus predominantly targets the respiratory tract, the widespread expression of angiotensin-converting enzyme 2 (ACE2) and its coreceptors such as transmembrane serine protease 2 (TMPRSS2) across human tissues enables it to affect multiple organ systems, including the endocrine glands (1–3). Since the first report by Bossoni et al. (4) describing severe hypocalcemia in COVID-19 patients, hypocalcemia has been recognized as a prominent biochemical abnormality associated with the disease (5). Importantly, several studies have shown that many patients fail to mount an appropriate compensatory increase in parathyroid hormone (PTH) despite profound hypocalcemia (6, 7). This phenomenon has often been broadly referred to as “functional hypoparathyroidism,” (7) which refers to patients without primary parathyroid disease who develop hypocalcemia and inappropriately low/low-normal PTH levels due to a specific pathophysiological state. In this state, parathyroid function can recover after correction of the underlying cause. Yet its clinical course and long-term outcomes remain poorly defined. At the same time, accumulating evidence suggests that SARS-CoV-2 may act as a trigger for autoimmune processes, either by exacerbating pre-existing endocrine disorders or by precipitating new-onset diseases (1). This observation is consistent with the broader understanding that viral infections can induce autoimmune responses through mechanisms such as molecular mimicry, bystander activation, and dysregulated immune signaling (8–10). Within the endocrine system, such processes may involve not only the thyroid (1–3) and pancreatic islets (1) but also the parathyroid glands (1, 3), raising the possibility that COVID-19 could contribute to persistent parathyroid dysfunction in susceptible individuals. However, the role of these two mechanisms in the parathyroid glands remains speculative and lacks direct evidence. Despite these concerns, case reports of COVID-19 associated hypoparathyroidism (HypoPT) remain rare, and most available descriptions lack systematic follow-up or detailed assessment of potential etiological mechanisms. Our literature search using PubMed, the China National Knowledge Infrastructure (CNKI), and Wanfang Data identified only a limited number of cases, the majority of which did not include long-term biochemical monitoring. Consequently, the relationship between SARS-CoV-2 infection and permanent HypoPT—a condition characterized by hypocalcemia due to insufficient PTH secretion that persists and does not resolve spontaneously—has not yet been clearly established. Current international guidelines provide a clear definition of permanence primarily for the most common form, postsurgical HypoPT: persistence of the condition for >12 months after surgery (11). In this report, we present a young woman who developed severe hypocalcemia after acute SARS-CoV-2 infection and was ultimately diagnosed with permanent HypoPT coexisting with Hashimoto’s thyroiditis (HT) after more than two years of follow-up. By combining this case with a comprehensive review of the literature, we aim to expand the clinical spectrum of COVID-19 associated HypoPT, highlight the potential role of autoimmune mechanisms, and underscore the importance of extended follow-up and etiological evaluation in patients with persistent calcium PTH axis dysfunction.

## Case presentation

A Chinese woman in her twenties, employed as a teacher, was admitted in 2023 with an 8-day history of generalized fatigue. Her symptoms began with unexplained fatigue, generalized pain, and paresthesia in the extremities, accompanied by fever, sore throat, nausea, abdominal distension, and a mild cough. She denied sputum production, dyspnea, chest pain, abdominal pain, diarrhea, vomiting, or other systemic complaints. At a local hospital, a SARS-CoV-2 antigen test returned positive (with no prior history of infection), and she was diagnosed with COVID-19. After taking oral ibuprofen alone, her fever, sore throat, and generalized pain resolved, but fatigue persisted. The paresthesia continued, and she subsequently developed intermittent carpopedal spasms (1–2 episodes per day, lasting about 30 minutes each, resolving spontaneously). During these episodes, she reported no dizziness, headache, seizures, facial asymmetry, dysarthria, or loss of consciousness. Two days before admission, her symptoms worsened, prompting her to present to the Emergency Department of a hospital in China. A repeat nasopharyngeal swab reverse transcription polymerase chain reaction showed that the SARS-CoV-2 result had turned negative. At that time, she reported only a mild occasional cough, with no fever, sputum production, or anorexia. Laboratory investigations (**Table 1**) revealed severe hypocalcemia. Serum calcium (Ca) was 4.57 mg/dL (reference range 8.46-10.10 mg/dL). Furthermore, arterial blood gas analysis demonstrated a critically reduced ionized calcium level (Ca²^+^ 2.36 mg/dL; reference range 4.61-5.17 mg/dL). Electrocardiogram (ECG) showed a corrected QT interval prolonged to 514 ms. She was treated with intravenous calcium and oral calcium acetate (0.6 g twice daily). Following supplementation, her tetany subsided, although she continued to experience generalized fatigue, numbness, and tightness in all four limbs. She denied muscle or joint pain, as well as visual or auditory disturbances, and was admitted for further evaluation.

**Table 1:** Laboratory data during COVID-19 infection and at select follow-up time points.

Measurement	Date										Ref. range
	Day -2	Day 0	Day 2	Day 6	15 d post-dc	~4 mo post-dc	~17 mo post-dc	~21 mo post-dc	~25 mo post-dc	~26 mo post-dc	
WBC (/μL)	3170	3580	/	/	6390	6970	7820	/	6480	/	3500-9500
Neutrophils (/μL)	1170	1850	/	/	3100	3560	4850	/	3290	/	1800-6300
Lymphocytes (/μL)	1630	1340	/	/	2820	2940	2560	/	2810	/	1100-3200
CRP (mg/L)	2.22	1.34	/	/	/	/	/	/	/	/	0-10
Hemoglobin (g/L)	127	124	/	/	129	134	136	/	136	/	115-150
pH	7.5	7.48	/	/	/	/	/	/	/	/	7.35-7.45
PaO_2_ (mmHg)	100	102	/	/	/	/	/	/	/	/	75-100
PaCO_2_ (mmHg)	28.8	36	/	/	/	/	/	/	/	/	35-45
SBE	-0.5	3.3	/	/	/	/	/	/	/	/	-3-3
Actual HCO_3_^-^ (mmol/L)	22.6	26.8	/	/	/	/	/	/	/	/	22-27
Standard HCO_3_^-^ (mmol/L)	24.8	27.6	/	/	/	/	/	/	/	/	22-27
Lactate (mmol/L)	2.3	0.9	/	/	/	/	/	/	/	/	0.5-2.2
Ca (mg/dL)	4.57	6.29	6.13	7.54	8.62	9.10	7.54	8.06	8.14	8.34	8.46-10.10
P (mg/dL)	5.67	4.83	5.61	5.33	5.33	5.54	5.33	5.73	5.57	4.86	2.63-4.68
Mg (mg/dL)	1.63	2.04	1.73	1.70	1.82	1.85	1.77	1.90	1.85	1.92	1.82-2.48
PTH (pg/mL)	11.3	/	9.4	/	<6.3	<6.3	14	11.2	7	/	18.5-88
25(OH)D (ng/mL)	/	12.06	/	/	24.96	35.92	28.48	/	29.61	29.41	20-100
Urea (mmol/L)	/	3.46	/	/	3.67	4.38	3.1	/	3.86	/	2.6-7.5
Creatinine (umol/L)	/	42	/	/	50	52	56	/	51	/	41-73
Albumin (g/L)	/	43.8	/	/	47.3	48.4	47.8	/	47.8	/	40-55
ALP (U/L)	/	60	/	/	52	49	49	/	65	/	35-100
Total Bilirubin (umol/L)	/	9	/	/	9.8	11.2	10.7	/	12.6	/	0-21
ALT (U/L)	/	42	/	/	8	9	10	/	11	/	7-40
AST (U/L)	75	32	/	/	15	17	18	/	16	/	13-35
LDH (U/L)	435	335	/	/	154	/	180	/	166	/	120-250
CK (U/L)	2050	1226	/	/	/	/	/	/	108	/	40-200
CK-MB (U/L)	59	37	/	/	/	/	/	/	11	/	0-25
Urinary Calcium (mmol/24h)	/	/	1.18	/	5.01	4.5	3.02	/	3.17	4.2	2.5-7.5
Urinary Phosphate (mmol/24h)	/	/	/	/	/	13.1	10.2	/	7.4	4.9	22.0-48.0
FT3 (pmol/L)	4.74	/	/	/	/	4.73	5.83	/	5.37	5.12	3.5-6.5
FT4 (pmol/L)	13.14	/	/	/	/	13.64	15.84	/	14.7	14.75	10.5-24.38
TSH (mIU/L)	2.493	/	/	/	/	2.67	2.317	/	3.076	2.243	0.55-4.78
TPOAb (IU/mL)	/	>1300	/	/	/	/	>1300	/	>1300	>1300	0-60
TgAb (IU/mL)	/	622.9	/	/	/	/	570	/	565.1	452	0-4.5
8 AM Cortisol (nmol/L)	/	/	620.8	/	/	/	/	/	435.4	/	117.7-686.6
8 AM ACTH (pg/mL)	/	/	21.55	/	/	/	/	/	26.305	/	7.2-63.4
GH (ug/L)	/	/	0.7	/	/	/	/	/	/	/	0.13-9.88
IGF-1 (ng/mL)	/	/	229	/	/	/	/	/	/	/	102-345
**Events**	**Emergency Department**	**Admission**		**Discharge**	**Some Follow-up Time Points**						

WBC, White Blood Cell; CRP, C-Reactive Protein; pH, Potential of Hydrogen; PaO_2_, Partial Pressure of Oxygen in arterial blood; PaCO_2_, Partial Pressure of Carbon Dioxide in arterial blood; SBE, Standard Base Excess; Actual HCO_3_^-^, Actual Bicarbonate; Standard HCO_3_^-^, Standard Bicarbonate; ALP, Alkaline Phosphatase; ALT, Alanine Aminotransferase; AST, Aspartate Aminotransferase; LDH, Lactate Dehydrogenase; FT3, Free Triiodothyronine; FT4, Free Thyroxine; TSH, Thyroid-Stimulating Hormone; TPOAb, Thyroid Peroxidase Antibody; TgAb, Thyroglobulin Antibody; d, days; mo, months; ~, approximately; dc, discharge.

All time points are presented relative to the date of admission (Day 0) or discharge to protect patient privacy. The bolded text in the last row indicates the core clinical events in the patient's disease timeline.

The patient provided results from a routine physical examination performed more than one month prior to admission. Thyroid ultrasound at that time showed diffusely reduced and heterogeneous echogenicity in both lobes, while thyroid function was normal (thyroid autoantibodies were not assessed). ECG was essentially normal. Of note, her annual health check-ups during the preceding two years had not included electrolyte or PTH testing. Her past medical history was otherwise unremarkable. She had no prior surgery or trauma, no history of neck irradiation, and no tobacco or alcohol use. She was unmarried, nulliparous, and reported a regular menstrual cycle. Family history was negative: both parents were healthy and non-consanguineous, and her elder sister was also healthy. She denied any family history of tuberculosis, malignancy, epilepsy, diabetes, hypertension, thyroid disease, autoimmune disorders, calcium-phosphorus metabolism abnormalities, or other hereditary diseases, and specifically denied any known genetic disorders.

On admission, the patient was afebrile with a temperature of 36.3 C, pulse rate of 94 beats/min, respiratory rate of 21 breaths/min, and blood pressure of 93/68 mmHg. Oxygen saturation was 98% on room air. She measured 1.62m in height and weighed 47kg. The patient was alert and oriented, with clear speech. Examination of the skin, hair, and dentition revealed no abnormalities. The thyroid gland appeared non-enlarged on inspection; palpation demonstrated a soft texture without tenderness. Pulmonary auscultation was clear, with no dry or wet rales. Neurological examination revealed a negative Chvostek’s sign but a positive Trousseau’s sign. Cardiovascular, abdominal, and other systemic examinations were unremarkable. A repeat ECG performed at admission was normal.

Relevant laboratory investigations and arterial blood gas analysis on room air are summarized in **Table 1**. Additional tests, including blood amylase, electrolytes (potassium, sodium, and chloride), serum iron, high-sensitivity troponin I (hs-cTnI), N-terminal pro–B-type natriuretic peptide (NT-proBNP), serum thyroglobulin, thyrotropin receptor antibody, routine stool and urine analyses, and coagulation studies (including D-dimer), were all within normal limits.

Given the presence of severe hypocalcemia with an inappropriately low PTH level and concomitant hyperphosphatemia, the patient was clinically diagnosed with HypoPT. Treatment was initiated with intravenous and oral calcium supplementation, together with oral vitamin D_3_ and calcitriol.

**Figure 1:**
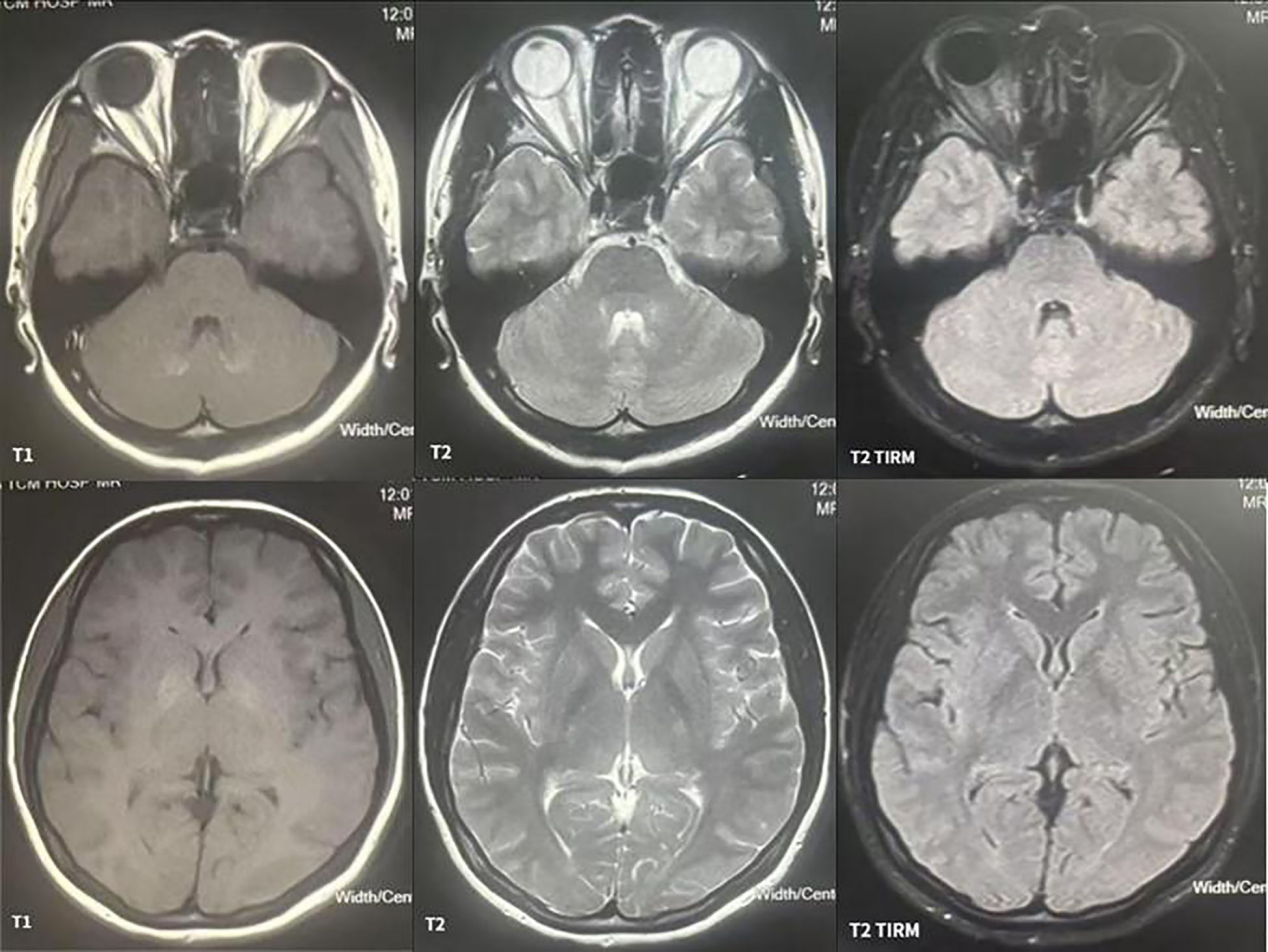
Non-contrast cranial MRI of the obtained on admission was unremarkable.

Following symptomatic improvement, thyroid and parathyroid ultrasound showed diffusely heterogeneous thyroid parenchyma with small patchy hypoechoic areas. The parathyroid glands showed no morphological abnormalities. Additional imaging including cardiac, abdominal, urinary tract, and bilateral cervical vascular ultrasonography showed no abnormalities. A non-contrast cranial MRI was unremarkable (**Figure 1**). Ophthalmological evaluation, including slit-lamp examination and adjunctive testing, also revealed no significant abnormalities.

Given the patient’s young age at onset and concomitant HT, an extended panel of laboratory tests was performed. Results showed that blood glucose, insulin, adrenocorticotropic hormone (ACTH), cortisol, growth hormone (GH), insulin-like growth factor (IGF), a complete sex hormone panel, calcitonin, antinuclear antibody, and extractable nuclear antigen (ENA) antibody profile were within normal limits. Due to the patient’s refusal and technical limitations, genetic testing and specific autoimmune marker testing were not performed.

With treatment, her generalized fatigue and paresthesia improved rapidly, and tetany in the hands and feet did not recur. After a 6-day hospital stay, she was discharged on the following regimen: Calcium Carbonate and Vitamin D_3_ Tablets 600 mg orally three times daily, Calcitriol Soft Capsules 0.25 μg orally three times daily, and Potassium Magnesium Aspartate Tablets one tablet orally three times daily. She was advised to undergo long-term follow-up with regular monitoring of electrolytes, renal function, and related parameters, with medication doses adjusted as needed.

**Figure 2:**
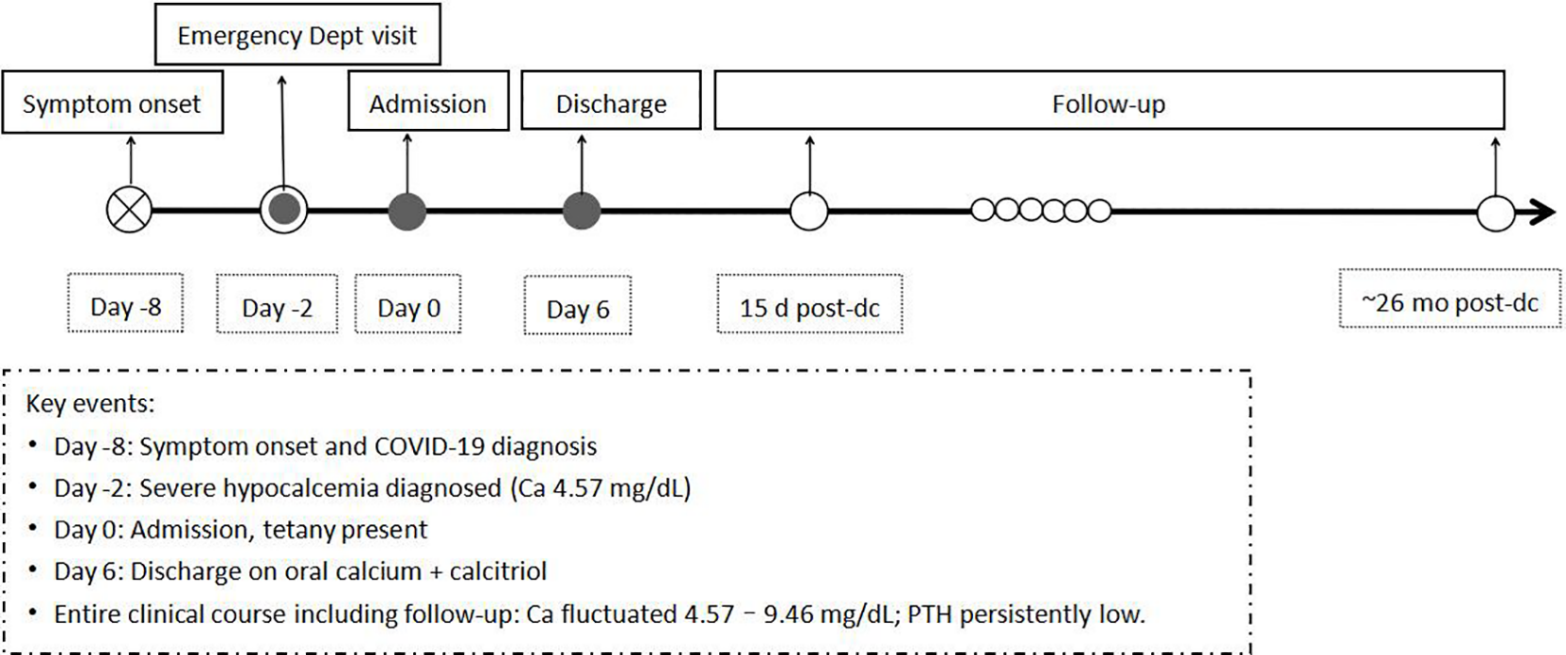
Clinical timeline of the case, illustrating key events from symptom onset and COVID-19 diagnosis (day -8) to the final follow-up (approximately 26 months post-discharge). Serum calcium levels fluctuated between 4.57 and 9.46 mg/dL throughout the course, with persistently low PTH.

After discharge, her elemental calcium supplementation ranged from 900 to 1800 mg per day, calcitriol was titrated between 0.25 and 0.75 μg per day, and Potassium Magnesium Aspartate Tablets were maintained at 2–3 tablets daily. To date, she has undergone 29 serum electrolyte assessments and 7 PTH monitoring sessions, which revealed calcium fluctuating between 4.57 and 9.46 mg/dL, phosphorus (P) between 4.24 and 6.22 mg/dL, and magnesium (Mg) between 1.56 and 2.14 mg/dL (representative results are presented in **Table 1**). Potassium, sodium, and chloride remained consistently within normal ranges. No spontaneous recovery of PTH secretion was observed. Follow-up urinary tract and thyroid ultrasounds showed no significant change from prior, and the patient has reported feeling well during follow-up without significant discomfort. A timeline of the clinical course is presented in **Figure 2**.

## Discussion

Since Bossoni et al. first reported severe hypocalcemia in COVID-19 patients in April 2020 (4), numerous studies have confirmed that hypocalcemia is highly prevalent among COVID-19 cases and is significantly associated with disease severity and prognosis (3, 5–7). Pal et al. (5) further demonstrated that even non-severe patients exhibited a prevalence of hypocalcemia as high as 67%, with serum calcium levels markedly lower than those of matched healthy controls, supporting the view that hypocalcemia may represent an intrinsic biochemical feature of COVID-19.

More strikingly, despite the high prevalence of vitamin D deficiency and hypocalcemia in patients with COVID-19, secondary hyperparathyroidism has been observed in only 14.3%–20.5% of patients (6, 7). Whether SARS-CoV-2 impairs endogenous PTH/vitamin D regulation, leading to insufficient PTH secretion, remains to be verified. Potential mechanisms may include: (1) inflammatory stimuli and respiratory alkalosis increasing calcium-sensing receptor (CaSR) sensitivity; (2) direct viral invasion of parathyroid tissue; (3) vascular injury, thrombosis, hypoxia, and immune dysregulation; and (4) concomitant magnesium deficiency (3, 5, 7). This case is consistent with this constellation, with severe hypocalcemia, inappropriately low PTH, respiratory alkalosis due to hyperventilation, vitamin D deficiency, and mild hypomagnesemia. Interestingly, the presence of hyperphosphatemia contrasts with Pal’s study, which found reduced phosphorus in non-severe patients (5).

HypoPT itself is rare, typically presenting with hypocalcemia, hyperphosphatemia, and low PTH, and can lead to diverse complications (11). While surgery remains the most common cause, approximately 25% of cases are nonsurgical, involving autoimmune destruction, genetic variants, radiation, metabolic derangements, or infiltrative diseases (11, 12). Autoimmunity is particularly important, and even idiopathic HypoPT may have an autoimmune basis (12, 13). Classically, vitamin D deficiency presents with hypocalcemia, hypophosphatemia, and secondary hyperparathyroidism (11, 14). Furthermore, the persistently low PTH in our patient, despite profound hypocalcemia, cannot be explained by mild hypomagnesemia alone. With >2 years of follow-up showing persistently suppressed PTH despite supplementation, a diagnosis of permanent HypoPT may be more appropriate than “functional hypoparathyroidism” (7).

To analyze this case in a broader context, we conducted a systematic search for literature on COVID-19-associated HypoPT via PubMed (without restrictions on language, country, or publication date) and by manually reviewing the references of relevant articles. The search strategy was as follows: “SARS-CoV-2” (or “COVID-19”) and “Hypoparathyroidism” (or “Hypocalcemia”/”Parathyroid hormone”). We excluded reviews, guidelines, editorials, commentaries, and articles with incomplete key information such as serum calcium or PTH levels. Additionally, we searched the CNKI and Wanfang Data and found no relevant Chinese reports. As of May 2025, a total of 14 published case reports were identified. Including the present case, this brings the total to 15 cases (see **Tables 2A**, **B**). The median age was 53 years (range 14-82), and 47% were female. Most patients presented with seizures, paresthesia, psychiatric symptoms, or dysarthria, while three were asymptomatic. In 87% (13/15), manifestations developed within two weeks of COVID-19 diagnosis, with the latest at five weeks. Disease severity was evenly distributed among mild, severe, and critical forms. Three patients died during hospitalization. Among survivors, eight required ongoing calcium/vitamin D therapy, but only seven cases had documented follow-up: three at 1 month, one at 6 months, two near 1 year, and our case beyond 2 years (representing the longest documented follow-up duration among all cases). Of these, four continued requiring supplementation; only one achieved full biochemical recovery. Functional classification distinguished 8 cases of decompensated pre-existing HypoPT and 7 cases of new-onset disease.

**Table 2A:** Clinical characteristics of the present case and those described in the literature on COVID-19-associated HypoPT.

Variable	Author [Refs]	Age (yrs)/sex	c-Ca (mg/dL)[RR]	P (mg/dL)[RR]	Mg (mg/dL)[RR]	PTH (pg/mL)[RR]	25 (OH) D (ng/mL)[RR]
Case 1	Bossoni et al. (4)	72/F	4.75 [ND]	5.2 [ND]	ND	10 [15-65]	8 [ND]
Case 2	Pla et al. (15)	76/M	6.14 [8.6-10.2]	5.4 [2.5-4.5]	2.42 [1.6-2.4]	23 [15-65]	22 [>30]
Case 3	Elkattawy et al. (16)	46/M	9.2 [ND]	5.8 [2.4-4.7]	1.9 [1.8-2.5]	8 [12-88]	7 [30-100]
Case 4	Demir et al. (17)	68/F	6.1 [ND]	7.8 [ND]	ND	2.8 [ND]	5.36 [ND]
Case 5	Bonnet et al. (18)	82/M	6.97 [ND]	2.91 [ND]	1.99 [ND]	9.06 [15-65]	44.4 [ND]
Case 6	Dianatfar et al. (19)	44/F	6.2 [8.5-11]	5.7 [2.7-4.5]	2 [1.9-2.5]	<3 [11-67]	33.1 [30-100]
Case 7	Grigoravičius et al. (20)	39/M	4.4 [8.4-10.2]	6.6 [2.3-4.7]	1.41 [1.6-2.6]	3.14 [15-68]	15.1 [30-40]
Case 8	Georgakopoulou et al. (21)	53/M	6.9 [8.6-10.2]	4.7 [2.5-4.5]	ND	11.7 [12-65]	38.4 [>30]
Case 9	Irisson-Mora et al. (22)	63/F	4.8 [8.6-10.2]	8.3 [2.7-4.5]	2.1 [1.7-2.5]	19.7 [11.3-82.7]	31 [20-100]
Case 10	Azanjac et al. (23)	62/M	5.05 [ND]	ND	ND	4.2 [ND]	10.08 [ND]
Case 11	Markov et al. (24)	63/F	5.2 [8.5-10.3]	4.9 [2.3-4.5]	1.2 [1.4-2.5]	13 [15-65]	16 [30-80]
Case 12	Bitew et al. (25)	48/M	2.6 [8.6-10]	11.2 [2.5-4.5]	ND	12.2 [12.2-64.9]	7 [ND]
Case 13	Selva et al. (26)	14/M	3.6 [8.3-10.6]	10.2 [2.4-5.1]	1.82 [1.6-2.6]	14.7 [14.9-56.9]	21.74 [ND]
Case 14	Inoue et al. (27)	34/F	6.6 [8.8-10.1]	3.2 [2.7-4.6]	1.8 [1.7-2.6]	25.9 [15-65]	#
Case 15	Present Patient	20s/F	4.57 [8.46-10.10]	5.67 [2.63-4.68]	1.63 [1.82-2.48]	11.3 [18.5-88]	12.06 [20-100]

Refs, References; yrs, years; M, male; F, female; RR, Reference range attached to the original text; ND, Not documented in the original text.

c-Ca, Corrected total calcium (mg/dL) = measured total calcium (mg/dL)+0.8×(4.0−serum albumin(g/dL)).

#, The measured biomarker was 1,25(OH)_2_D. The patient’s level was 37 pg/mL (reference range: 20-60).

The units for the biochemical indicators in the table have been standardized.

**Table 2B:** Clinical characteristics of the current case and those described in the literature on COVID-19-associated HypoPT.

Variable	Course of COVID-19 infection	Onset	Symptoms/signs related to HypoPT	Type of dysfunction	The discharge treatment plan	Follow-up status
Case 1	Mild	Day 3 of COVID-19 symptoms	Acute-onset dysarthria; perioral paresthesia	Decompensated pre-existing HypoPT	Needs: Ca/calcitriol supplements.	ND
Case 2	Severe	During hospitalization for COVID-19 treatment	Perioral paresthesia; upper extremity paresthesia; positive Trousseau sign	Decompensated pre-existing HypoPT	ND	ND
Case 3	Critical	Month 2 of COVID-19 hospitalization	None,incidental finding	New-onset HypoPT	Requires administration of calcium, vitamin D, and sevelamer	1-month post-discharge: Normal Ca. Persistently low PTH and high P.
Case 4	Critical	Day 3 of confirmed COVID-19 hospitalization	Tonic-clonic convulsion	Decompensated pre-existing HypoPT	Died	/
Case 5	Severe	Day 5 of COVID-19 symptoms	None,incidental finding	New-onset HypoPT §	Discontinue calcium and calcitriol supplementation	1-month post-discharge:normal Ca/P/PTH.
Case 6	Severe	During the first to second week post-COVID-19 diagnosis	Tonic-clonic seizure; depressed mood	New-onset HypoPT	ND	ND
Case 7	Mild	On day 3 post-COVID-19 diagnosis	Tonic-clonic seizure; resting limbs tremor; dry skin and onycholysis; delirium	Decompensated pre-existing HypoPT	Needs: Ca/Mg/active vitamin D supplements.	ND
Case 8	Severe	Day 10 of COVID-19 symptoms	None,incidental finding	New-onset HypoPT	ND	1-month post-discharge: normal Ca/P, no PTH reported.
Case 9	Critical	Day 8 of COVID-19 symptoms	Convulsion with loss of alertness	Decompensated pre-existing HypoPT	Died	/
Case 10	Critical	During presentation of acute COVID-19 infection	Extrapyramidal signs with cerebellar dysfunction and anarthria	Decompensated pre-existing HypoPT	Died	/
Case 11	Severe	During presentation of acute COVID-19 infection	Leg cramps and finger numbness	New-onset HypoPT	Needs: Ca/Mg/vitamin D supplements.	ND
Case 12	Critical	During presentation of acute COVID-19 infection	Tonic-clonic rhythmic movement of the upper and lower extremities; spontaneous facial twitching; positive Trousseau sign; confusion, dysarthria	Decompensated pre-existing HypoPT	Needs: Ca/vitamin D/calcitriol supplements.	11-month post-discharge: normal Ca/P, no PTH documented, but right-sided facial twitching persisted.
Case 13	Mild	Day 4 of COVID-19 symptoms	Tonic-clonic seizures with carpopedal spasms, perioral numbness, distal limb tingling	New-onset HypoPT	Needs: Ca/calcitriol supplements.	6-month post- COVID: PTH remains low.
Case 14	Mild	During presentation of the second acute COVID-19 infection	Tetany	Decompensated pre-existing HypoPT	Needs: Ca/active vitamin D supplements.	Medications tapered off post-discharge. At 1-year follow-up: normal Ca and PTH, P not reported.
Case 15	Mild	During presentation of acute COVID-19 infection	Paresthesia in extremities, intermittent limb convulsions, positive Trousseau sign	New-onset HypoPT	Needs: Ca/Mg/vitamin D supplements.	Multiple follow-ups over 2+ years post-discharge: Persistently low PTH.

ND, Not documented in the original text.

§, In December 2019, the patient’s serum calcium level was measured at 8.22 mg/dL, but albumin was not simultaneously tested for correction. Given the high prevalence of hypoalbuminemia in elderly patients, this hypocalcemia result requires cautious interpretation. Furthermore, after calcium and vitamin D supplementation were discontinued at discharge, a one-month post-discharge re-examination showed that serum levels of calcium, phosphorus, and PTH had all returned to normal. In conclusion, the patient had developed new-onset HypoPT at that time, but subsequent follow-up confirmed that it was a transient event.

Notably, four distinctive cases were identified from the follow-up records of new-onset HypoPT patients and were classifiable into two groups based on their outcomes. In group 1, two patients (Case 5 and Case 8, aged 82 and 53 years) had normalized serum calcium and phosphorus levels at the 1-month post-discharge follow-up. In group 2, two patients (Case 13 and Case 15, both under 30 years old) still exhibited abnormal serum phosphorus and PTH levels at follow-up more than six months after COVID-19 recovery. This aligns with guideline recommendations suggesting that genetic testing may be of reference value for non-surgical HypoPT with young onset (11). More intriguingly, patients in Group 1 had “severe” COVID-19, yet their corrected total serum calcium levels (6.97 and 6.9 mg/dL, respectively) and 25-Hydroxyvitamin D [25(OH)D] levels (44.4 and 38.4 ng/mL, respectively) were relatively high. In contrast, patients in Group 2 had only “mild” COVID-19, but their corrected total serum calcium levels (3.6 and 4.57 mg/dL, respectively) and 25(OH)D levels (21.74 and 12.06 ng/mL, respectively) were significantly lower. Contrary to the previously held consensus that “patients with severe COVID-19 exhibit lower calcium and vitamin D levels,” the present finding highlights unresolved questions regarding the role of vitamin D status.

Beyond calcium-phosphate homeostasis, vitamin D also modulates innate and adaptive immunity and regulates inflammatory responses (28). Vitamin D deficiency is not only a significant risk factor for SARS-CoV-2 infection and adverse outcomes of COVID-19 (3), but also plays an important role in the pathogenesis of autoimmune diseases (29). It is known that viral infections can trigger autoimmunity through molecular mimicry (10), and accumulating evidence supports the involvement of autoimmunity in the pathogenesis of COVID-19-related endocrine dysfunction (1–3, 30, 31). The patient in this case had no pre-existing systemic diseases or family history. Although there is a clear temporal association between SARS-CoV-2 infection and HypoPT, the baseline levels of serum calcium, phosphorus, PTH, and vitamin D prior to infection remain unknown. Therefore, whether the condition represents subclinical or pre-existing HypoPT unmasked by infection, stress, or vitamin D deficiency remains speculative at this stage.

Another feature of this case was coexisting HT, diagnosed by ultrasound abnormalities and positive thyroid autoantibodies. The coexistence of multiple autoimmune endocrine disorders increases the risk of autoimmune polyglandular syndrome (APS) (32). Nearly half of APS patients test positive for thyroid autoantibodies without overt clinical thyroid disease (32). Given the marked heterogeneity of APS presentations and the potential emergence of new disease components or even phenotypic changes over disease course (33), its diagnosis remains highly challenging. APS-1, in particular, is caused by mutations in the AIRE gene, typically presents in childhood, and features the classic triad including HypoPT (32, 34). However, although HT is the most common organ-specific autoimmune disease, its predictive value for the subsequent development of other autoimmune conditions is generally considered to be far lower than that of rare diseases such as Addison’s disease (35). Therefore, in the present case, APS can only be considered as a theoretical possibility. Long-term follow-up, repeated autoantibody screening and genetic evaluation, as well as genetic counseling, are recommended (12, 32, 33, 35).

Rarely, Riedel’s thyroiditis has been described in conjunction with HT and HypoPT (36), thought to reflect systemic fibrotic or autoimmune processes (37). In our patient, however, the thyroid was soft, non-enlarged, and sonographically typical for Hashimoto’s, with markedly elevated autoantibodies, making Riedel’s disease unlikely. Nonetheless, careful longitudinal monitoring of thyroid function and imaging is advisable.

HypoPT-related hypocalcemia may also cause muscle injury. Dai et al. (38) reported nonspecific histological muscle changes in idiopathic HypoPT, where serum calcium <1.3 mmol/L correlated with creatine kinase (CK) elevations >10-fold. Mechanisms include tetany-induced muscle enzyme release and altered membrane permeability (38). Our patient experienced muscle discomfort with CK elevation, while creatine kinase-myocardial band (CK-MB), hs-cTnI, and NT-proBNP remained normal, consistent with skeletal rather than cardiac origin. CK normalized after calcium correction, echoing prior reports (14, 38). Because even asymptomatic patients may have subclinical muscle involvement, routine CK monitoring should be considered (38). Among published cases, one also reported mild CK elevation (18).

This case of permanent HypoPT with HT following COVID-19 expands the clinical spectrum of COVID-19 related endocrine sequelae and contributes long-term follow-up data to a very limited pool of literature. Several limitations should be acknowledged: (1) inflammatory cytokines were not measured during the acute phase; (2) no chest imaging was performed, as the patient declined ionizing radiation, and magnetic resonance imaging rather than computed tomography was used for cranial evaluation, which may have reduced sensitivity for calcifications; and (3) genetic testing and specific autoimmune marker assays were not available.

## Conclusions

SARS-CoV-2 may affect the endocrine system through both direct viral infection and secondary immune responses, but its involvement in parathyroid dysfunction remains uncertain and is supported mainly by case reports. The manifestations of HypoPT are heterogeneous, and baseline calcium-PTH data prior to SARS-CoV-2 infection are often unavailable, which limits causal inference. Available evidence, including this case, raises the possibility that COVID-19 might play a role in triggering new-onset HypoPT or revealing subclinical disease, and may, in some cases, be linked to hypocalcemic crisis. It may be reasonable to consider extended follow-up for patients who develop hypocalcemia with an inadequate compensatory PTH response after infection, particularly for those with young onset, no surgical history, and a mismatch between the severity of hypocalcemia and that of the infection. Autoimmune marker assessment and genetic testing may aid in clarifying etiology and guiding management.

## Data Availability

The original contributions presented in the study are included in the article/supplementary material. Further inquiries can be directed to the corresponding author.
